# A Decision Support System for Diagnosis of COVID-19 from Non-COVID-19 Influenza-like Illness Using Explainable Artificial Intelligence

**DOI:** 10.3390/bioengineering10040439

**Published:** 2023-03-31

**Authors:** Krishnaraj Chadaga, Srikanth Prabhu, Vivekananda Bhat, Niranjana Sampathila, Shashikiran Umakanth, Rajagopala Chadaga

**Affiliations:** 1 Department of Computer Science and Engineering, Manipal Institute of Technology, Manipal Academy of Higher Education, Manipal 576104, India; krishnaraj.chadaga1@learner.manipal.edu; 2 Department of Biomedical Engineering, Manipal Institute of Technology, Manipal Academy of Higher Education, Manipal 576104, India; niranjana.s@manipal.edu; 3 Department of Medicine, Dr. TMA Hospital, Manipal Academy of Higher Education, Manipal 576104, India; shashikiran.u@manipal.edu; 4 Department of Mechanical and Industrial Engineering, Manipal Institute of Technology, Manipal Academy of Higher Education, Manipal 576104, India; chadaga.r@manipal.edu

**Keywords:** COVID-19, clinical markers, deep learning, explainable artificial intelligence, machine learning

## Abstract

The coronavirus pandemic emerged in early 2020 and turned out to be deadly, killing a vast number of people all around the world. Fortunately, vaccines have been discovered, and they seem effectual in controlling the severe prognosis induced by the virus. The reverse transcription-polymerase chain reaction (RT-PCR) test is the current golden standard for diagnosing different infectious diseases, including COVID-19; however, it is not always accurate. Therefore, it is extremely crucial to find an alternative diagnosis method which can support the results of the standard RT-PCR test. Hence, a decision support system has been proposed in this study that uses machine learning and deep learning techniques to predict the COVID-19 diagnosis of a patient using clinical, demographic and blood markers. The patient data used in this research were collected from two Manipal hospitals in India and a custom-made, stacked, multi-level ensemble classifier has been used to predict the COVID-19 diagnosis. Deep learning techniques such as deep neural networks (DNN) and one-dimensional convolutional networks (1D-CNN) have also been utilized. Further, explainable artificial techniques (XAI) such as Shapley additive values (SHAP), ELI5, local interpretable model explainer (LIME), and QLattice have been used to make the models more precise and understandable. Among all of the algorithms, the multi-level stacked model obtained an excellent accuracy of 96%. The precision, recall, f1-score and AUC obtained were 94%, 95%, 94% and 98% respectively. The models can be used as a decision support system for the initial screening of coronavirus patients and can also help ease the existing burden on medical infrastructure.

## 1. Introduction

The SARs-CoV-2 pandemic began in late 2019 after the virus emerged in Wuhan, China. It was unlike other coronaviruses, such as SARS and MERS, which only caused mild symptoms [1]. SARS-CoV-2 turned out to be a global catastrophe causing approximately six million fatalities [2]. It is the greatest epidemic to have affected humanity since the Spanish flu in 1918 [3]. Eventually, vaccines were developed to prevent the severe symptoms of the virus. COVID-19 vaccines such as AstraZeneca, Pfizer, Covaxin, Covax, Moderna, Sinopharma and others were administered to people worldwide. The vaccine doses seem to prevent the severe symptoms caused by the virus, and the death rates have dropped considerably [4]. 

Cough, fever, shortness of breath and myalgia are mild symptoms commonly reported in COVID-19 patients [5]. It can be challenging to correctly diagnose coronavirus patients because their symptoms resemble those of the common cold and flu. The R-PCR test is widely used for diagnosing this potent virus. However, these tests are known to misdiagnose patients in a number of instances. False-negative results have also become extremely common [6]. Other limitations include higher turnaround time, lower sensitivity, and high equipment costs. Ribonucleic acid (RNA) sequences must also be genetically conserved. Therefore, it is crucial to find an alternative COVID-19 diagnosis method which is robust, readily available, efficient and accurate. The virus has also been detected using several other techniques such as X-rays, ultrasound, computerized tomography scans, and voice-based analysis [7].

Machine learning has been extensively used to assist the healthcare domain in the present era. AI can improve a doctor’s decision-making using mathematical models and visualization techniques. It also reduces the likelihood of physicians becoming fatigued due to excess consultations. The advancement of AI and relevant clinical datasets about various diseases are primarily responsible for the developments mentioned above [8]. The abundance of COVID-19 data has significantly increased due to computer platforms and data storage improvements. This gives medical professionals and researchers a rare chance to concurrently investigate variables impacting patient diagnosis and build innovative testing techniques for COVID-19 detection.

Blood test results are readily available and less expensive than some of the other modalities. Markers such as neutrophils, lymphocytes, white blood cells, monocytes, basophils and others have shown a correlation with COVID-19 [9]. Various studies have proved that blood markers can be used with artificial intelligence to diagnose COVID-19 [10,11]. These markers have also been used to predict COVID-19 severity in advance since they vary drastically before the onset of severe symptoms [12]. Several studies, some of which are discussed below, have used blood markers and AI to diagnose coronavirus infection.

Rahman et al. [13] developed a Sars-CoV-2 detection system using laboratory markers. Seven public datasets were used for testing and validation. A nomogram-based screening methodology called “QCovSML” was designed to predict COVID-19. An AUC of 96.1% was obtained for the validation dataset. Fang et al. [14] designed a lightweight model for COVID-19 diagnosis. Multiple laboratory markers containing blood tests, epidemiological parameters and patient demographics were utilized. The proposed classifier obtained a maximum accuracy of 97.17%. According to the study, the most critical parameters are fever, sore throat and cough. Rostani et al. [15] used an explainable method for COVID-19 diagnosis. The final model obtained an accuracy, f1-score, sensitivity, specificity and AUROC of 90%, 79%, 72%, 93% and 93%, respectively. According to the random forest explainable artificial intelligence (XAI) technique, the most important features were platelets, eosinophils, white blood cells, lymphocytes and hemoglobin. Thinoteo et al. [16] used XAI for COVID-19 detection in another study. Several clinical markers were utilized, including platelets, red blood cells, lymphocytes, monocytes and others. The logistic regression model obtained a maximum accuracy of 90%. According to SHAP, the most important markers were basophils, eosinophils, leukocytes, monocytes, lymphocytes and platelets. However, most of the studies used machine learning to diagnose COVID-19 from healthy patients. Further, most research has either used SHAP or LIME for model explainability. Deep learning algorithms were also not utilized in many studies.

The contemporary ML-based technologies’ explainability and interpretability should also be considered because they may make it more difficult to employ these technologies in healthcare systems. The reasoning behind a diagnosis by an ML classifier must be understandable since the doctor should be able to explain the information to their patients. This highlights why most AI models used in the healthcare industry primarily serve as prototypes that allow clinicians to ignore the classifier output before making a clinical decision [17]. Some doctors are reluctant to use ML models that are difficult to understand, comprehend and trust. Through model approximation, rule-based generation, local/global explanations and enhanced feature visualization, explainable AIs (XAI) attempt to explain the predictions made by the ML classifiers. Visualization models such as Shapley additive explanations (SHAP), local interpretable model explainer (LIME), QLattice and eli5 have been utilized for model interpretability [18].

In this study, we use ML and DL techniques to distinguish COVID-19 from other similar infections, such as flu and influenza, using clinical and laboratory markers. The study also provides a trustworthy COVID-19 diagnostic method that can be applied generally in healthcare institutions. Quick screening for COVID-19 is vital for resource utilization and the planning of treatments. The contributions of our article are given below:

- We have collected our own COVID-19 dataset containing patient data of COVID-19 and non-COVID-19 influenza-like illness (ILI) patients from two Manipal hospitals in India. Prior ethical clearance has also been obtained to conduct this study.
- The statistical tool “JAMOVI” has been used to conduct a descriptive statistical analysis of the data.
- The grey wolf optimizer has been utilized for feature selection to choose the most essential clinical markers.
- Different ML algorithms have been tested to predict COVID-19 diagnosis. The algorithms have been further stacked on multiple levels to improve accuracy. Deep learning models such as deep learning networks (DNN) and one-dimensional convolutional neural networks (1D-CNN) have also been utilized to test model effectiveness.
- XAI techniques such as SHAP, LIME, Eli5 and QLattice have made the models more understandable and interpretable.
- Further discussion about COVID-19 diagnosis using important clinical markers is presented.

As of today, no studies exist which use four XAI techniques to validate COVID-19 diagnosis from non-COVID-19 ILI using clinical markers. The remainder of the article is as follows: materials and methods are discussed in Section 2; results and discussion are explained in Section 3; and future directions and the conclusion are discussed in Section 4. 

## 2. Materials and Methods

### 2.1. Dataset Description

The dataset was collected from two hospitals: Kasturba Medical College and Dr. TMA Pai hospital in India. Prior ethical clearance has been obtained with the identification number IEC:613/2021 from Kasturba Medical College and Dr. TMA Pai hospital to conduct this research. The data were collected from March 2021 to December 2021 during the second wave of the COVID-19 pandemic. Blood test reports of 1169 patients tested for COVID-19 were considered for the study. All the patients were above 18 years of age. Each patient displayed symptoms such as cough, myalgia and fever. The standard RT-PCR test was used for COVID-19 diagnosis. Out of 1169 patients, 270 patients were identified as COVID-19 negative. The number of attributes chosen was 24, including the results of the RT-PCR test (label). Most of the attributes were continuous (22 attributes). The results for the parameters ‘Gender’ and ‘RT-PCR’ were categorical in nature. The attributes are clearly described in Table 1. 

### 2.2. Dataset Preprocessing

During initial data preprocessing, missing values are filled using various statistical measures such as mean, median and mode. Categorical values are encoded, and redundant features are dropped. The data are also scaled to prevent a dataset from having a high range (difference between the maximum value and minimum value). Data balancing is also performed if there is a considerable difference between the two classes. While collecting data, we have chosen patients who had undergone most tests to ensure a minimum number of missing values. The median was used to impute the remaining missing values for continuous variables since it does not get affected by outliers. “Gender” is the only categorical variable in our dataset, and it did not contain any null values. The “Jamovi” application was used to conduct descriptive statistical analysis. This is an open-source statistical software which is used by researchers to conduct descriptive and inferential statistics [19]. Table 2 describes some of the statistical measures used in this research. 

The performance suffers significantly when there is a large disparity among data points. Additionally, the models prioritize features with higher values irrespective of the units used. Data scaling is vital to get good results in machine learning. Standardization and normalization are the two ways to scale the data [20]. Normalization converts all the data point values between zero and one based on the maximum and minimum values. The equation for normalization is given below:

(1) $$N_{norm}=\frac{N-min\;(N)}{\max\;\left(N\right)-min\;(N)}$$

where *N* is a value from attribute *N*, min (*N*) is the minimum value of the attribute *N* and max (*N*) is the highest value of the attribute *N*. The “MinMaxScalar” library is used to implement normalization in python. When attribute values are standardized, the standard deviation is set to one and the feature points are clustered around its mean (mean = 0). The formula for standardization is given below:

(2) $$N_{standard}=\frac{N-Mean\;(N)}{S.D\;(N)}$$

where *N* is the value of the feature *N* and *Mean* (*N*) and *S.D* (*N*) are the mean and the standard deviation, respectively. In this study, standardization was used to perform scaling since it handles outliers effectively. The “Standard Scalar” library is used to perform standardization in python.

**Figure 1:**
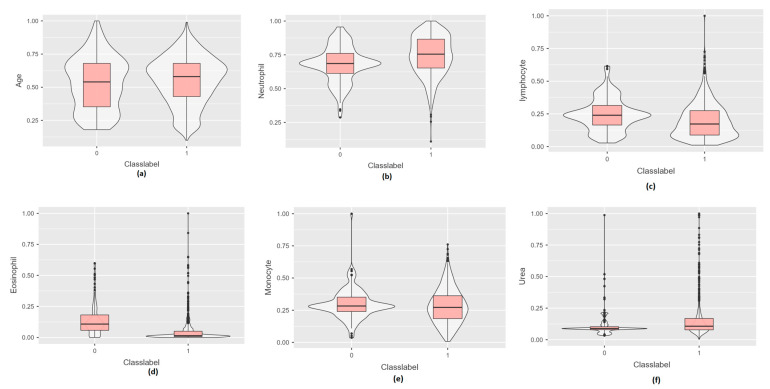
Violin plots used to represent COVID-19 and non-COVID-19 patients. (0—non COVID-19 ILI patients, 1—COVID-19 patients). (**a**) Age (**b**) Neutrophil (**c**) Lymphocyte (**d**) Eosinophil (**e**) Monocyte (**f**) Urea.

**Figure 2:**
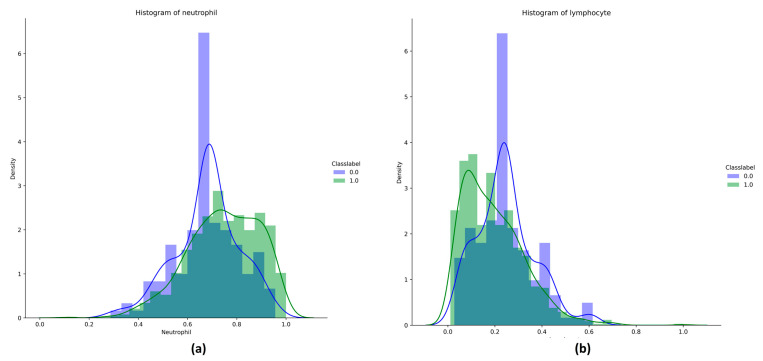
Histograms of neutrophil and lymphocyte counts for COVID-19 and non-COVID-19 patients. (0—non-COVID-19 ILI patients, 1—COVID-19 patients). (**a**) Neutrophil (**b**) Lymphocyte.

To visualize the data better, violin plots and histograms were used as depicted in Figure 1 and Figure 2. It can be seen that the mean age of COVID-19 patients is higher than non-COVID-19 ILI patients. The neutrophil count is also slightly higher in COVID-19 patients. However, the lymphocyte count decreases in COVID-19 patients. It is also observed that eosinophil levels are slightly higher in ILI COVID-19 patients. There is not much variation in the monocyte count between the two classes. Further, urea levels are slightly elevated in COVID-19 patients. It can also be seen that there are outliers present in some of the attributes present in the dataset. The IQR technique was used to handle the outliers in our study. Here, using the IQR values, we have capped the extreme values above the upper whisker value to the value of upper whisker (Quartile 3, Q3) and similarly capped the extreme lower value to the lower whisker (Quartile 1, Q1). 

**Figure 3:**
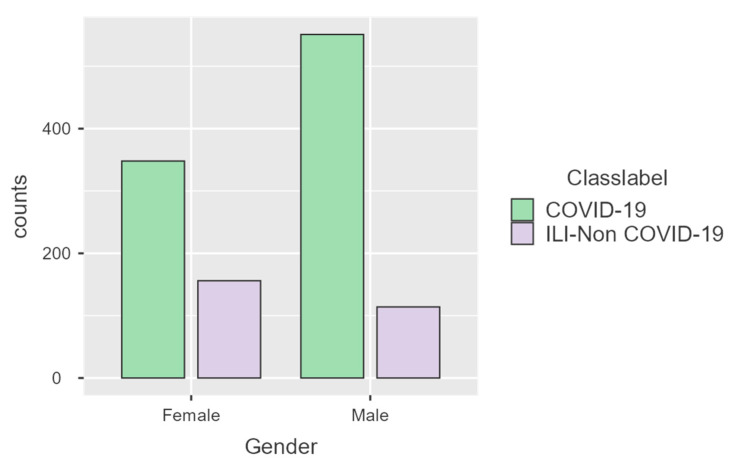
Bar graph depicting male and female population present in the dataset. (0—non-COVID-19 ILI patients, 1—COVID-19 patients).

Gender was the only categorical variable present in the dataset. The dataset had 665 male patients and 504 female patients. The COVID-19 negative ILI class had 114 male patients and 156 female patients. The COVID-19 class had 551 male patients and 348 female patients. The gender count for each class is pictorially depicted using a bar graph in Figure 3. 

Categorical features must be encoded into numbers before model training. This step is essential because a number of the classifiers do not handle text data. There are various encoding techniques, such as one-hot encoding, label encoding, binary encoding, hash encoding and others. The one-hot encoding method was used in this research since it prevents models from assuming more significant numbers [21] by assigning a new attribute to each value present in the label. Each attribute created will have only binary values (0/1). The dataset was split in the ratio of 80:20 (training and testing). 

Medical data are often imbalanced, causing the data to be proportionally distorted. The number of non-COVID-19 ILI patients was 270 and the number of COVID-19 patients was 899. The dataset had to be balanced because the classifiers favor the class with higher instances. In this study, a variant of the synthetic minority oversampling technique (SMOTE) called borderline-SMOTE was used to balance the training dataset [22]. Borderline-SMOTE uses the KNN classifier to generate a synthetic dataset. The testing dataset was not balanced to safeguard the reliability of the classifiers to predict new data.

### 2.3. Grey Wolf Optimizer for Feature Selection

Feature selection is used to choose the most important features and eliminate the unnecessary ones. Data have been generated in massive numbers as a result of the significant spread of current technology and intelligent systems. After the completion of feature selection, concerns such as redundancy and noise are significantly minimized [23]. In this research, we used the grey wolf optimizer to perform feature selection [24]. As the name suggests, it is a nature-inspired metaheuristic algorithm based on the behavior of wolves. The wolves live in packs which follow a strict hierarchical structure. Each wolf is ranked based on its power and strength. They are divided into alphas, betas, gammas and omegas. The pack is led by the alpha (both male and female) at the highest level of hierarchy. The alphas are those who lead the pack and take decisions. Beta wolves are the next in command and they help in supporting the alpha wolves. They also help maintain discipline in the pack. The delta wolf is ranked lower than the beta wolf. They are powerful but lack self-confidence and leadership skills. The omegas are the least powerful in the hierarchy. They are generally old and help in taking care of the wolf pups. The wolf hierarchical order is not just about power and aggression. It also helps weaker members (baby, old and injured wolves) who are unable to find prey. Besides social hierarchy, they also have distinct hunting approaches. A few wolves isolate their prey while others attack it after tiring it. 

Mirjalili et al. [25] developed the grey wolf optimization (GWO) technique. GWO employs natural predatory mechanisms such as searching, cornering and hunting. Each wolf represents a prospective solution, with the prey representing the optimum solution. GWO uses fewer parameters compared with other feature selection algorithms. It is also highly accurate ,known for its quick execution and is easy to implement. GWO has already been used in several machine learning healthcare applications [8,9]. 

In this research, we used the GWO wrapper class provided by Jingwei Too to perform feature selection [26]. Twenty features were chosen by the GWO algorithm. The features chosen by the algorithm are as follows: urea, albumin, neutrophil, protein, potassium, AST, sodium, basophil, hemoglobin, lymphocyte, NLR, hematocrit, monocyte, TWBC, age, T. bilirubin, D. bilirubin and creatinine.

### 2.4. Machine Learning Terminologies and Pipeline

Machine learning is the field of science that understands how machines learn without being explicitly programmed by prior training models before actual testing. As the name implies, it gives the computer characteristics that make it more human-like. This technology is used in many domains, such as engineering, medicine, life sciences, and marketing. Machine learning is grouped into three classes: (a) supervised learning, (b) unsupervised learning, and (c) reinforcement learning. In supervised ML, the training data are labelled. These models learn from the data and predict accurate results when a given dataset has been efficiently trained. The following are the list of ML classification algorithms used in this study: random forest, logistic regression, decision tree, KNN, Adaboost, catboost, lightgbm, xgboost and custom stacked models. Deep learning algorithms such as DNN and 1D-CNN were also considered.

Stacking can combine different classification or regression models [13]. The two well-known ensemble modelling techniques are bagging and boosting. Bagging enables the averaging of several comparable models with significant variance to reduce entropy, boosting creates numerous incremental algorithms to reduce bias while minimizing variance and stacking uses an alternative approach. Exploring the space of various designs for the same issue is the goal of stacking. The concept involves approaching a learning problem with several classifiers that can grasp a portion of the issue but not the entire problem space. Multiple distinct learners can be built in order to generate an interim prediction. Afterward, a new model is included that picks up the same label from intermediate predictors. The final model is stacked on other models, hence the name. This improves the accuracy and is often better than any individual model. The result is also trustworthy, because the models are built using various heterogeneous classifiers. We have used multiple stacking models in this study.

All the models were tested under a five-fold cross validation method. Cross-validation is a mathematical technique used to assess the competence of ML classifiers [13,14]. It is often used in applied AI to evaluate and choose a model for a specifically given criterion since it is simple to grasp, implement, and produces lower bias than other methods. Cross validation is a resampling method used to examine machine learning models on a small sample of data. A value called k is used to decide the number of subgroups the dataset must be split into. When a specific value for k is decided, it may be substituted for k in the model’s reference, such as k=5 for five-fold cross-validation. The data are shuffled before the actual process. This method is largely used to quantify a model’s skill in correctly classifying new data. It uses a tiny proportion of testing data to assess a model’s performance in generating predictions and decisions on data that was not utilized during the training phase. The results obtained during k-fold cross-validation are summarized with the average scores. 

We also used the grid search tuning technique to choose the best hyperparameters [15]. Any ML algorithm performance can be improved significantly using hyperparameter tuning techniques. As a result, determining the ideal hyperparameters will assist in achieving the highest-performing model. Hyperparameters can be chosen using different techniques, such as grid search, random search, manual search, and Bayesian optimization. In this research, we have used the grid search technique to identify the best hyperparameters to increase the model output. This technique finds the total performance for each combination of all the available hyperparameters and associated values and then selects the best value for the hyperparameters and uses cross-validation before tuning the hyperparameters. The grid search function is easily available in python under the scikit-learn class “model_selection”. 

**Figure 4:**
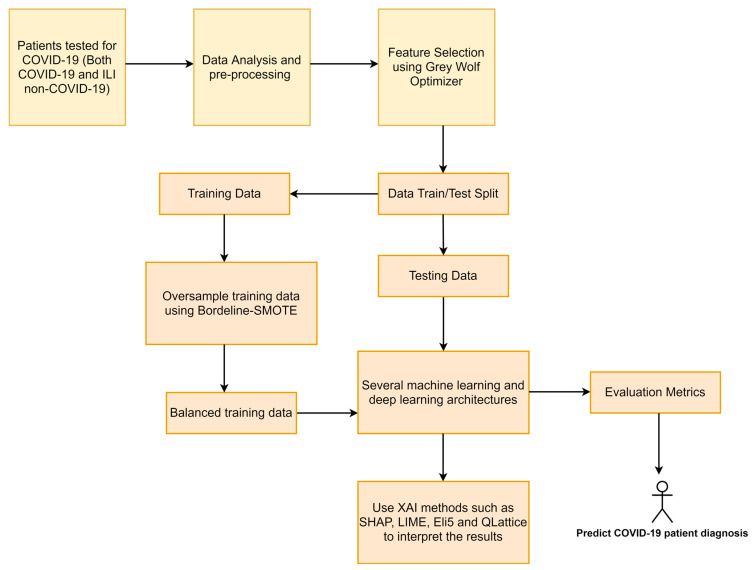
Machine learning pipeline to diagnose COVID-19 using clinical markers.

The output generated by the ML algorithms can now be understood and trusted by human users because of a set of procedures and techniques known as explainable artificial intelligence [17,18]. This describes the model and indicates the impact and biases. It contributes to the definition of model correctness, transparency, fairness, and decision-making outcomes. AI is developing each day, and it is of utmost importance for humans to understand and comprehend how a machine learning algorithm arrives at a result. The entire calculating procedure is transformed into what is known as a ‘black box’, which is harder to decipher. The existing data create these ‘black box’ models. XAI has a lot of advantages, improving the explainability of the model and making the classifiers more precise. It also helps researchers from various backgrounds to understand the interpretability of the machine learning model. In this research, three techniques were utilized: SHAP, LIME, QLattice and Eli5. The pipeline of the ML architecture is depicted in Figure 4.

## 3. Results and Discussion

### 3.1. Performance Measures

The classifiers were evaluated using measures such as accuracy, precision, recall, f1-score and AUC in this study. The metrics are explained below.

*Accuracy*: The number of accurately predicted COVID-19 and ILI COVID-19 negative cases (in percentage). It is described using the equation given below.

(3) $$Accuracy=\frac{True\;positives\;+\;True\;negatives}{True\;positives\;+\;True\;negatives\;+\;False\;positives\;+\;False\;negatives}$$

*Precision*: The proportion of ILI COVID-19 negative cases that are correctly predicted. The number of false positive cases are low when the model obtains high precision. It is calculated using the equation below.

(4) $$Precision=\frac{True\;positives\;+\;True\;negatives}{True\;positives\;+\;False\;negatives}$$

*Recall*: The proportion of COVID-19 cases accurately predicted and the number of false negative cases when the model obtains a high recall. It is calculated using the below equation.

(5) $$Recall=\frac{True\;positives}{True\;positives\;+\;False\;negatives}$$

*F1-score*: This considers both precision and recall. F1-score is important when false positive and false negative cases are equally important. It is calculated using the below equation.

(6) $$F1-score=\frac{2\;*\;(Precision\;*\;Recall)}{\left(Precision+Recall\right)}$$

*ROC curve*: This can be used to examine the association between false positive values and true positive values. The true positive values are plotted against the false positive values. The area under this curve is called AUC. When the AUC is higher, the model is more efficient.

### 3.2. Model Evaluation Using Machine Learning and Deep Learning

**Figure 5:**
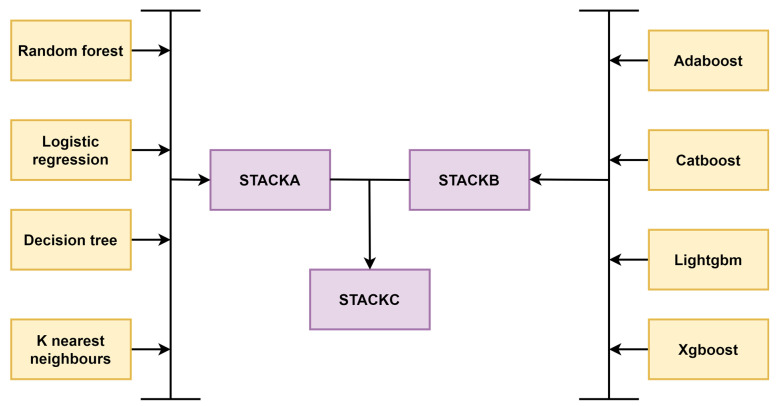
Custom stacked architecture to predict COVID-19 from other ILI patients.

In this research, COVID-19 patients were screened using clinical markers and machine learning. This is essential because many other diseases have similar symptoms to those of COVID-19. Furthermore, these approaches have the potential to reduce the tremendous load already existing on healthcare facilities. All the models were run using python with the help of the anaconda library. Prior to model training, the Borderline-SMOTE was used to balance the training dataset. Feature scaling and data balancing were undertaken for the training dataset. ML models such as random forest, logistic regression, decision tree and K nearest neighbors were tested in the beginning. To improve the results, the above models were stacked to form the custom ensembled algorithm. This stacked model was named ‘STACKA’. Further, boosting algorithms such as adaboost, catboost, lightgbm and xgboost were also tested. The above classifiers were ensembled to form the custom ‘STACKB’ model. To obtain the best performance, ‘STACKA’ and ‘STACKB’ were further ensembled. The combined classifier ‘STACKC’ was used for COVID-19 prediction. The custom stacked multi-level architecture model is described in Figure 5. 

**Figure 6:**
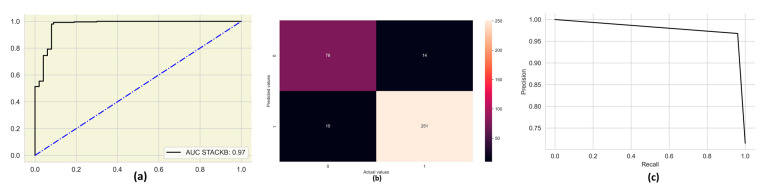
AUC’s and confusion matrices obtained by STACKA, STACKB and STACKC (Stacked models). (**a**) AUC (**b**) Confusion matrix (**c**) Precision-Recall curve.

We used python libraries such as scikit learn, matplotlib, seaborn, numpy and pandas to run the models. For deep learning, libraries such as tensorflow and keras have been utilized. Among the baseline models, random forest performed very well with an accuracy, precision, recall, f1-score and AUC of 94%, 94%, 89%, 91% and 99%, respectively. The stacked model (STACKA) obtained an accuracy of 90%. Among the boosting algorithms, the lightgbm and xgboost obtained the best results. The lightgbm AND xgboost obtained an accuracy of 96%. The stacked model (STACKB) was able to obtain an accuracy, precision, recall, f1-score and AUC of 96%, 95%, 95%, 95% and 99%, respectively. The combined STACKC model obtained an accuracy of 96%. STACKC will be considered for prediction since it is a combination of various classifiers. The results of the heterogenous classifiers are summarized in Table 3 and the AUC curves and confusion matrices of the models are described in Figure 6. Hyper parameters included are described in Table 4. 

We also tested deep learning models. Deep neural network (DNN) and 1D-CNN were the two classifiers utilized. A DNN consists of input layer, output layer and many hidden layers [27]. DNN’s are capable of modelling complex non-linear patterns. A DNN’s principal function is to handle user inputs, execute progressively sophisticated computations on the data, and output results which can help us when making a decision. 

For DNN, we constructed a neural network with six different layers. The input layer consisted of 21 neurons (input features). The hidden layers consisted of 12, 9, 7 and 4 neurons. The architecture used for DNN is described in Table 5. “ReLU” was the activation function utilized for the input and hidden layers. For the output layer, the sigmoid activation function was utilized. “Adam” served as the neural network’s optimizer. Binary cross entropy was the chosen loss function. A learning rate of 0.0001 was used to obtain optimal results. The number of epochs was set to 1000 and the batch size was set to 10. For training and testing, the data were divided in a ratio of 80% to 20%. The DNN was able to obtain good results in our study. The accuracy, precision, recall, f1-score and AUC obtained were 87%, 80%, 86%, 83% and 90%, respectively. 

**Figure 7:**
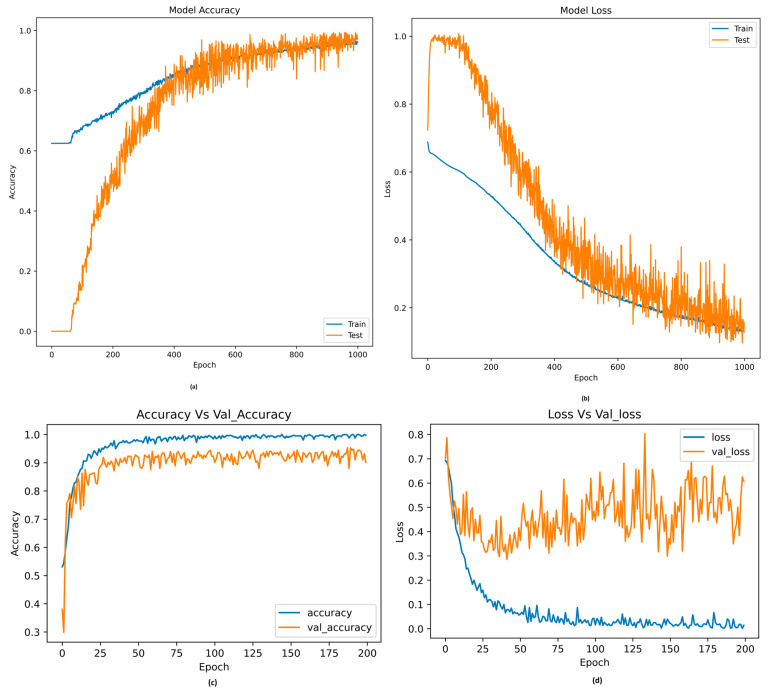
Accuracy and loss curves obtained by the DNN and 1D-CNN models. (**a**) Accuracy curve of DNN. (**b**) Loss curve of DNN. (**c**) Accuracy of 1D-CNN. (**d**) Loss curve of 1D-CNN.

Further, we used the 1D-CNN model for COVID-19 diagnosis [28]. CNN classifiers were initially used for image classification, in which the algorithm goes through a process called feature extraction by taking a 2D-array as input. A similar approach can be applied to one-dimensional or tabular data. The advantage of using a 1D-CNN is that they extract information from raw data and do not need domain expertise. The architecture of a 1D-CNN classifier is described in Table 6. The model consists of nine layers which include Conv1D, Maxpooling1D, dropout, flatten and dense layers. Adam was the optimizer utilized. The binary cross entropy was used to measure the loss. The number of epochs was set to 200 and the batch size was set to 10. The model performed extremely well with an accuracy of 90%. The precision, recall and f1-score obtained were 86%, 89%, 88% and 93%, respectively. The results obtained by DNN and 1D-CNN model are summarized in Table 7 and the accuracy and loss curves are described in Figure 7. In our study, the algorithms performed very well at the task of distinguishing COVID-19 from other diseases with similar symptoms.

### 3.3. Explainable Artificial Intelligence (XAI) to Interpret Results

The diagnosis made by the classifiers will have a substantial impact in healthcare decision making. Automation and computerization of different operations and activities have been brought about by technological breakthroughs. As a result, algorithms that are very precise, clear and understandable have been chosen. In the complex realm of medicine, an interpretable model enhances a medical professional’s ability to confirm the claimed diagnosis. It is also critical to evaluate the application’s output before reaching a final therapeutic decision. Furthermore, for a system to be durable, feature assessments that depend on a variety of variables are crucial. In this study, four XAI models were used: (a) SHAP, (b) LIME, (c) Eli5, and (d) QLattice. These feature importance methods explain the reasoning behind the predictions made by the ML model. The XAI models were used to interpret the results obtained by the random forest model since they obtained good results. 

**Figure 8:**
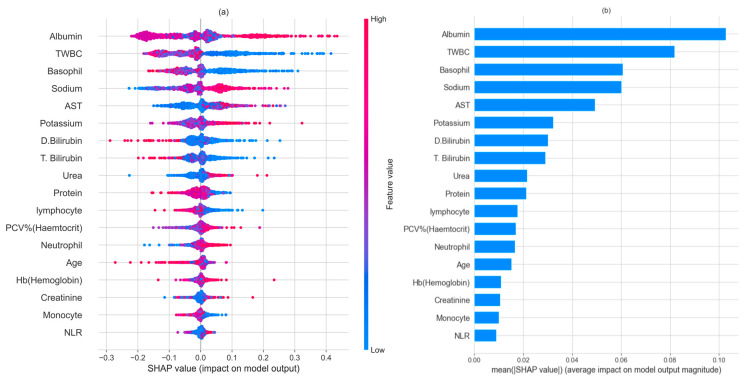
SHAP plots for model explainability. (**a**) bee swarm plot, (**b**) bar plot.

Game theory and probability are the foundations of SHAP [29]. For instance, the coefficients of a model assess the overall importance of each attribute, though there can be errors because the values are scaled. The local relevance of the property and how it changes with different levels are not considered by the coefficients. Consequently, SHAP can be a huge assistance in understanding tree-based models. The bee swarm plot and the mean bar plot generated by the SHAP model are shown in Figure 8. A hyperplane in Figure 8a divides the ILI non-COVID-19 and COVID-19 classes. The attributes are ranked from most important to least important. A lower value is denoted by the color blue, and larger values are denoted by the color red. SHAP claims that the presence of albumin, TWBC, basophil, sodium and AST are crucial in distinguishing COVID-19 from other similar infections. AST levels tend to increase for COVID-19 patients in this study. TWBC and basophil levels decrease for COVID-19 patients. Other important attributes include potassium, D. bilirubin, T. bilirubin, urea and protein. The mean effect of SHAP values on the classifiers output magnitude is shown in Figure 8b.

**Figure 9:**
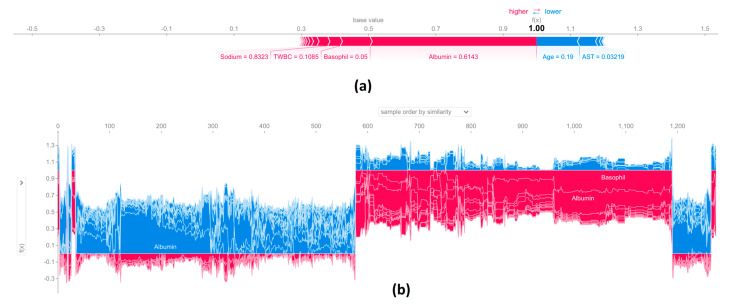
SHAP force plots for model explainability. (**a**) Force plot for a particular instance, (**b**) force plot for all instances.

**Figure 10:**
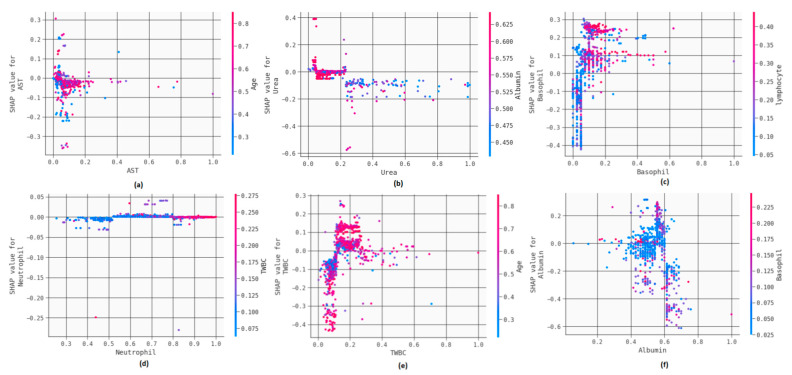
SHAP dependence plots of some clinical markers. (**a**) AST, (**b**) urea, (**c**) basophil, (**d**) neutrophil, (**e**) TWBC, and (**f**) albumin.

A SHAP model can improve the predictions generated for a specific patient by using a force plot. Figure 9a describes a force plot for a patient predicted to be COVID-19 positive. Features on the left side (red color) predict a positive COVID-19 diagnosis and attributes on the right side (blue color) predicts a negative COVID-19 diagnosis. Near the line separating the red from the blue are the elements that have a greater influence on the score. The bar’s width measures the feature’s influence. Though some of the attributes indicate a COVID-19 negative diagnosis, important attributes such as albumin, basophil and TWBC indicate a positive COVID-19 diagnosis. Hence the red (more important attributes) shift the blue attributes (less important features). Figure 9b indicates the force plot for all the instances. SHAP dependence plots are very useful for identifying the relationship between two different variables. In the dependence plot, the datapoints lying between attributes are analyzed. Figure 10 describes the dependence plots of a number of variables.

**Figure 11:**
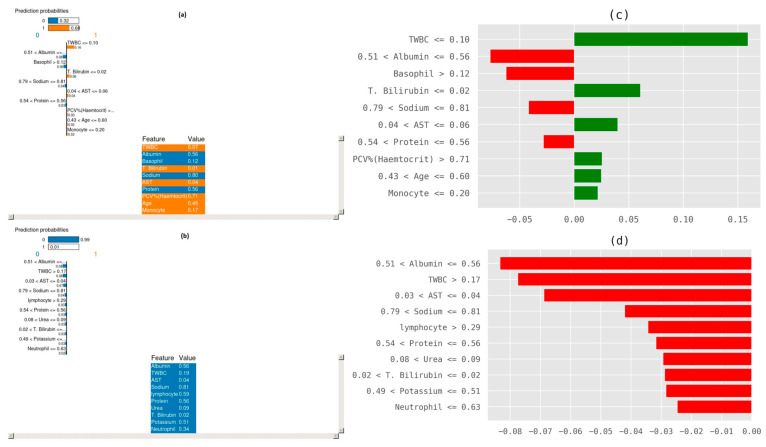
LIME interpretation for model output. (**a**) Positive COVID-19 prediction (**b**) Negative COVID-19 prediction (**c**) Positive COVID-19 prediction (**d**) Negative COVID-19 prediction.

LIME can also comprehend the results of the ML classifier [30]. Attributes that call for explanations are initially picked and, after the model’s predictions have been made, the initial data are modified to understand the model’s outcomes. The new data points must be allocated weights based on the proximity of their relevant occurrences. Numerous combinations are obtained by the models and are used for training. Finally, an explanation is provided and an interpretation is given for the predictions. The LIME models are explained in Figure 11. Figure 11a,c indicate the LIME interpretation for a COVID-19 positive patient. It can be seen that attributes such as TWBC, T. bilirubin and AST indicate a positive COVID-19 diagnosis. When each attribute predicts a different diagnosis, the weights (importance) of the attributes are considered. Figure 11b,d indicate the LIME interpretation for a COVID-19 negative patient. It can be seen that all attributes indicate a COVID-19 negative diagnosis.

**Figure 12:**
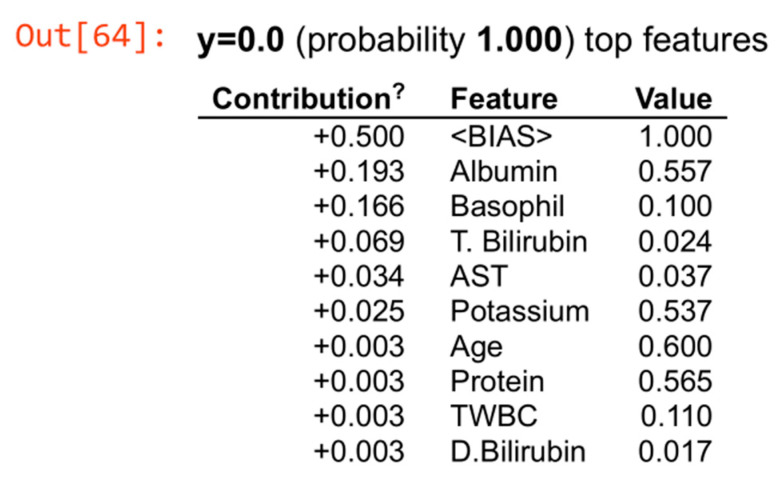
Eli5 model to understand model predictions.

Another XAI method to analyze and justify predictions is Eli5 [31]. It visualizes and troubleshoots predictions using API’s. This enables researchers to comprehend various classifiers when seeking to understanding predictions. Figure 12 describes the explainability provided by the Eli5 model. From the figure, it can also be seen that albumin, basophil, T. bilirubin, AST, potassium, age, protein, TWBC and D. bilirubin are the most important parameters. Eli5 also considers the bias parameter while explaining the model. 

**Figure 13:**
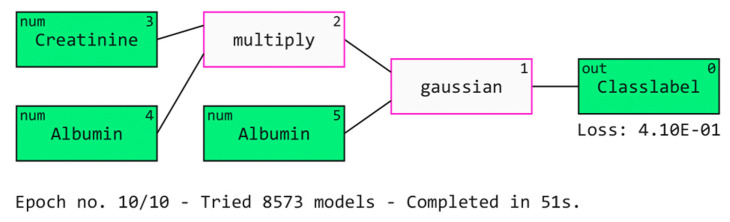
The use of QGraph to explain model predictions.

A transparent architecture called QLattice is comparatively new in ML [32]. This offers a thorough explainability to the blackbox concept seen in conventional models. QLattice looks through thousands of potential models before settling on the one that best fits the problem. The user must first set up a few parameters, including input properties and other variables. In this method, the attributes are known as registers. The generated model is called a “QGraph”. The graph consists of edges and nodes. Each edge is assigned a weight, and an activation function is assigned to each node. When the QGraph is fully trained, critical information about the attributes are generated. QLattice is implanted using the “Feyn” library in python. Figure 13 represents a QGraph. From the figure, it can be seen that the model considers albumin and creatinine as the most important attributes. This model also uses the “multiply” and “gaussian” function to interpret results. The transfer function of the XAI model is explained using Equation (7):

(7) $$logreg(2.5-2.8e^{-26000.0{\left(0.56-Albumin\right)}^{2}{\left(Creatinine+0.44\right)}^{2}-3.7{\left(0.59-Albumin\right)}^{2}})$$

### 3.4. Further Discussion

In this research, ML and DL algorithms were used to distinguish COVID-19 from other diseases with similar symptoms using a set of clinical markers. The dataset consisted of 1169 patients from two hospitals in India who had undergone the RT-PCR test. Statistical analysis was performed using “JAMOVI” to understand the trends in the data. The grey wolf optimizer technique was used for feature selection. To better understand the results, four XAI techniques were utilized. The ML models can be used as an initial decision support system to screen COVID-19 patients.

AST is a liver enzyme which elevates when there is a liver infection. Our data confirm that COVID-19 patients have high AST levels, which have been reported in numerous articles [33,34]. In COVID-19 patients, TWBC is seen to decrease, according to the research. Numerous investigations have already established that COVID-19 causes leukopenia [35,36]. Low TWBC levels increase the risk of infection. Albumin levels were comparatively higher in SARS-CoV-2 patients [37]. Lower albumin levels indicate damage to the liver. Further, decreased Basophil count was observed in COVID-19 patients. Basopenia has been observed in COVID-19 patients according to many studies [38,39]. Patients with COVID-19 had slightly higher sodium and potassium levels. Neutrophil count was elevated for COVID-19 patients and lymphocyte count decreased for COVID-19 patients. This is a general trend observed in coronavirus patients, according to many studies [40,41]. Neutrophil and lymphocyte count are also monitored to predict severity [42]. Urea and creatinine levels were slightly higher in COVID-19 patients, a finding which aligns our research with other similar studies [43,44]. Elevated levels of the above markers indicate a damage to the kidneys. Protein and monocyte levels were comparatively lower in COVID-19 patients. These were some of the observations made from this study. 

There is no individual marker which can diagnose COVID-19 patients. However, a combination of markers and AI can be used to predict COVID-19 [45]. Several studies have already used AI to diagnose COVID-19 using hematological and clinical markers. Rikan et al. [46] used AI techniques to detect COVID-19 from routine blood tests. Three clinical datasets were considered for their study and, by using Pearson, Spearman, and Kendall’s coefficients, feature selection was carried out across seven ML and four DL models. The DNN obtained a maximum accuracy of 92.11%. Barbosa et al. [47] developed an intelligent system for COVID-19 diagnosis, wherein 24 blood parameters were considered and an overall accuracy of 95.15% was obtained. In another study, XAI techniques were used to understand a COVID-19 diagnosis [16]. SHAP and LIME were the two XAI methods used in this research and the most important parameters were eosinophils, white blood cells and leukocytes. An AUC of 87% was obtained by the best models. 

A stacked model was utilized by Rahman et al. [13] to diagnose COVID-19. In their study, seven open datasets were compared and a stacking model obtained an accuracy of 91.44%. Fang et al. [14] designed a weight learning mechanism for COVID-19 detection. Multiple clinical and laboratory datasets were considered and a maximum accuracy of 97.17% was obtained by the best performing model. Rostami et al. [15] developed a novel XAI technique for COVID-19 diagnosis. The most important features according to this study were platelets, eosinophil, TWBC, lymphocytes, ALT and hemoglobin. Bartenschlager et al. [48] developed “COVIDAL” to diagnose COVID-19 in Germany, with 4000 patients considered for their study. The accuracies, sensitivities and specificities obtained were up to 90%. From the above studies, it is clear that machine learning and clinical markers can aid in the accurate detection of COVID-19. 

Machine learning generally takes less computational time and is faster, though deep learning algorithms are more accurate. If data preprocessing is not conducted, execution time can be faster, but the results are more reliable after data preprocessing. Ethics are also important in medical artificial intelligence. Validation of the models must also be performed.

However, there are several limitations to the study. The data used were collected from two hospitals in Manipal. For better generalizability, data from several geographical areas must be considered. The role of antibiotics before COVID-19 diagnosis was not considered. Antibiotics can change the levels of markers such as TWBC considerably. Combing modalities such as X-rays, CT-scans, MRI’s and ultrasounds should also be considered. GPU’s, which can decrease computational time, were not used in this study. Further, unsupervised machine learning and reinforcement techniques can also be considered. 

## 4. Conclusions

The COVID-19 pandemic turned out to be a fatal disaster causing millions of deaths worldwide. The RT-PCR test is widely used to diagnose COVID-19. However, the tests take a considerable amount of time and are also prone to incorrect outcomes. Hence, various other techniques such as, X-rays, clinical markers, CT scans and voice-based analysis have been used for COVID-19 diagnosis. In this research, clinical and laboratory markers were used to detect COVID-19 from other infections which cause similar symptoms. The data consisted of 1169 patients from Kasturba Medical College and Dr. TMA Pai hospital. Grey wolf optimizer was chosen for feature selection and 18 attributes were considered from the initial 24 attributes. A multi-level stacked ensemble classifier was developed to detect COVID-19 and this obtained an accuracy, precision, recall, f1-score and AUC of 96%, 94%, 95%, 94% and 98%, respectively. DNN and 1D-CNN models were also tested. To interpret model predictions, four XAI techniques were used. These were SHAP, LIME, Eli5 and QLattice. According to these, the most important markers are albumin, ALT, basophil and TWBC. The combination of these markers can be used to screen COVID-19 patients. The classifiers can be used as a decision support system to assist healthcare professionals.

In the future, datasets from different countries can be chosen to establish reliability and graphical processing units (GPU’s) can be used for faster execution of the model. Cloud-based models can be used to store data and model infrastructure. Further, other modalities, such as X-rays and CT scans, should also be considered. The dataset should also be expanded as deep learning models are more effective when the dataset is large.

## Figures and Tables

**Table 1:** Clinical markers present in the dataset.

Sl. No	Marker	Attribute Datatype	Description	Sl. No	Marker	Attribute Datatype	Description
1	Age	Demographic/Continuous	Age of a patient (In years)	13	Creatinine	Clinical/Continuous	It is an amino acid commonly found in muscles and brain. Higher levels of creatinine indicate damage to the kidney (mg/dL).
2	Gender	Demographic/Categorical	Gender of the patient (Male/ Female)	14	Sodium	Clinical/Continuous	Electrolytes which help body function by maintaining blood and volume. Higher levels of sodium can lead to hypertension (mmol/L).
3	Hemoglobin (Hb)	Clinical/Continuous	It carries oxygen to the organs of the body. It is a part of red blood cells (RBC) (gram/dL).	15	Potassium	Clinical/Continuous	Electrolytes which help body function by maintaining blood and volume. Lower levels of potassium can lead to hypertension (mmol/L).
4	Hematocrit	Clinical/Continuous	It indicates the proportion of RBC in blood (in %).	16	Total Bilirubin	Clinical/Continuous	It is a combination of direct and indirect bilirubin (mg/dL).
5	Total White blood cells (TWBC)	Clinical/Continuous	It fights infection and is a part of the immune system (10^3^/microliter).	17	Direct Bilirubin	Clinical/Continuous	RBC’s are broken down by the body, creating a chemical called bilirubin (mg/dL).
6	Neutrophil	Clinical/Continuous	A type of WBC. Higher levels of neutrophil indicate an infection (% -Differential count).	18	Aspartate transaminase (AST)	Clinical/Continuous	It is an enzyme present in the liver. Higher levels of AST indicate damage to the liver (IU/L).
7	Lymphocyte	Clinical/Continuous	A type of WBC. Lower levels of lymphocyte indicate an infection (% -Differential count).	19	Alanine transaminase (ALT)	Clinical/Continuous	It is an enzyme present in the liver. Higher levels of AST indicate damage to the liver (IU/L).
8	NLR (Neutrophil to Lymphocyte ratio)	Clinical/Continuous	Number of neutrophils per lymphocytes. Higher levels of NLR indicate an infection (Whole number).	20	Alkaline phosphatase (ALP)	Clinical/Continuous	It is an enzyme present in the liver. Higher levels of AST indicate damage to the liver (IU/L).
9	Monocyte	Clinical/Continuous	A type of WBC. Varying levels of monocyte indicate infection in the body.	21	Protein	Clinical/Continuous	Total protein present in our blood (g/dL).
10	Eosinophil	Clinical/Continuous	A type of WBC. Varying levels of monocyte indicate infection in the body. (% -Differential count)	22	Albumin	Clinical/Continuous	A protein present in blood. Lower levels can indicate damage to kidneys or liver (g/dL).
11	Hemoglobin A1c (HbA1c)	Clinical/Continuous	It reveals the median blood sugar over a period of two to three months. Higher levels of HbA1c indicate diabetes (In %).	23	Urea	Clinical/Continuous	It is a main component of urine and removes unnecessary nitrogen. Higher levels of urea indicate damage to the kidney (mg/dL).
12	Basophil	Clinical/Continuous	A type of WBC (% -Differential count).	24	RT-PCR test results	Clinical/Categorical	Results of the RT-PCR test (COVID-19 positive/COVID-19 negative)

**Table 2:** Descriptive statistics of the COVID-19 data collected.

Feature	Class Label	Mean	Median	SD	IQR	Range	Minimum	Maximum	25th	50th	75th
Age	ILI (COVID-19 negative)	52.711	54	19.929	32.75	82	18	100	35.25	54	68
	COVID-19 positive	55.108	58	17.8	25	81	18	99	43	58	68
Hb(Hemoglobin)	ILI (COVID-19 negative)	12.305	12.4	1.824	1.675	11.2	6.1	17.3	11.6	12.4	13.275
	COVID-19 positive	12.718	12.9	2.158	2.65	15	3.7	18.7	11.55	12.9	14.2
PCV%(Haemtocrit)	ILI (COVID-19 negative)	36.406	36.5	5.133	4.675	31.75	19.45	51.2	34.2	36.5	38.875
	COVID-19 positive	37.762	38	6.274	7.8	48.5	9	57.5	34.2	38	42
TWBC	ILI (COVID-19 negative)	8.497	7.95	4.316	2.425	33.9	1.2	35.1	6.55	7.95	8.975
	COVID-19 positive	8.449	6.5	5.995	5.1	58.8	0.2	59	4.9	6.5	10
Neutrophil	ILI (COVID-19 negative)	66.599	67.2	12.854	14.525	65.6	28.1	93.7	60.05	67.2	74.575
	COVID-19 positive	72.977	74	14.207	20.9	87.36	10.64	98	63.95	74	84.85
Lymphocyte	ILI (COVID-19 negative)	22.342	21.8	11.159	13.625	53.5	2.5	56	15	21.8	28.625
	COVID-19 positive	17.721	15.7	11.923	17	90	1	91	8	15.7	25
NLR	ILI (COVID-19 negative)	4.615	3	5.4	2.75	40	1	41	2	3	4.75
	COVID-19 positive	8.242	4	11.118	8	92	1	93	2	4	10
Monocyte	ILI (COVID-19 negative)	8.223	7.8	3.218	3.075	26.6	1	27.6	6.625	7.8	9.7
	COVID-19 positive	7.761	7.5	3.772	4.9	20.8	0.2	21	5.1	7.5	10
Eosinophil	ILI (COVID-19 negative)	1.99	1.5	1.904	1.725	8.3	0	8.3	0.8	1.5	2.525
	COVID-19 positive	0.698	0.2	1.355	0.6	13.9	0	13.9	0.1	0.2	0.7
Basophil	ILI (COVID-19 negative)	0.492	0.4	0.423	0.3	2.5	0	2.5	0.3	0.4	0.6
	COVID-19 positive	0.316	0.2	0.287	0.2	4	0	4	0.2	0.2	0.4
Urea	ILI (COVID-19 negative)	26.839	21.5	22.294	5.75	232	8	240	19.25	21.5	25
	COVID-19 positive	36.745	26	35.139	22	242.3	0.7	243	19	26	41
Creatinine	ILI (COVID-19 negative)	0.938	0.8	0.681	0.2	7.3	0.4	7.7	0.7	0.8	0.9
	COVID-19 positive	1.211	0.9	1.383	0.4	14.8	0.2	15	0.7	0.9	1.1
Sodium	ILI (COVID-19 negative)	133.911	135	5.163	3.75	30	112	142	132.25	135	136
	COVID-19 positive	135.526	136	5.531	7	56	111	167	132	136	139
Potassium	ILI (COVID-19 negative)	4.126	4.1	0.387	0.3	2.8	3.2	6	4	4.1	4.3
	COVID-19 positive	4.245	4.2	0.659	0.8	5.9	2.1	8	3.8	4.2	4.6
T. Bilirubin	ILI (COVID-19 negative)	0.716	0.5	1.127	0	12.2	0.2	12.4	0.5	0.5	0.5
	COVID-19 positive	0.695	0.5	1.129	0.38	21	0	21	0.32	0.5	0.7
D.Bilirubin	ILI (COVID-19 negative)	0.362	0.2	0.899	0	9.6	0.1	9.7	0.2	0.2	0.2
	COVID-19 positive	0.341	0.2	0.731	0.2	11.96	0.04	12	0.1	0.2	0.3
AST	ILI (COVID-19 negative)	46.719	33	62.99	0	589	10	599	33	33	33
	COVID-19 positive	55.941	39	65.605	36	900.8	0.2	901	26	39	62
ALT	ILI (COVID-19 negative)	41.648	35	34.909	2.375	257	9	266	33.375	35	35.75
	COVID-19 positive	46.095	32	58.912	30	696.5	3.5	700	20	32	50
ALP	ILI (COVID-19 negative)	95.622	89	44.133	0	469	35	504	89	89	89
	COVID-19 positive	95.135	81	62.826	39	880	5	885	65	81	104
Protein	ILI (COVID-19 negative)	7.021	7	0.414	0	3.1	5.9	9	7	7	7
	COVID-19 positive	6.893	7	0.685	0.6	9.2	3.2	12.4	6.6	7	7.2
Albumin	ILI (COVID-19 negative)	3.847	3.9	0.349	0	3.1	1.5	4.6	3.9	3.9	3.9
	COVID-19 positive	3.846	3.9	0.574	0.9	6.6	0.4	7	3.4	3.9	4.3
HbA1c	ILI (COVID-19 negative)	6.1	5.8	1.311	0	9.1	4	13.1	5.8	5.8	5.8
	COVID-19 positive	6.806	6.2	1.872	1.8	14.2	4	18.2	5.6	6.2	7.4

**Table 3:** Results obtained by ML classifiers.

Algorithm	Accuracy (%)	Precision (%)	Recall (%)	F1-Score (%)	AUC (%)
Random forest	94	94	89	91	99
Logistic regression	68	65	70	68	74
Decision tree	81	75	83	77	88
KNN	81	75	83	77	83
STACKA	90	85	90	87	96
Adaboost	94	91	94	92	95
Catboost	90	86	86	86	96
Lightgbm	96	94	95	94	98
Xgboost	96	95	93	94	99
STACKB	96	95	95	95	99
STACKC	96	94	95	94	98

**Table 4:** Hyperparameters chosen by the algorithms.

Algorithm	Hyperparameters Chosen
Random forest	{‘bootstrap’: True, ‘max_depth’: 110, ‘max_features’: 2, ‘min_samples_leaf’: 3, ‘min_samples_split’: 8, ‘n_estimators’: 300}
Logistic regression	{‘C’: 100, ‘penalty’: ‘l2’}
Decision tree	{‘criterion’: ‘gini’, ‘max_depth’: 40, ‘max_features’: ‘sqrt’, ‘min_samples_leaf’: 1, ‘min_samples_split’: 10, ‘splitter’: ‘best’}
KNN	{‘n_neighbors’: 1}
STACKA	{use_probas=True, average_probas=False, meta_classifier=Logistic Regresion}
Adaboost	{‘learning_rate’: 1.0, ‘n_estimators’: 300}
Catboost	{‘border_count’: 32, ‘depth’: 3, ‘iterations’: 250, ‘l2_leaf_reg’: 3, ‘learning_rate’: 0.03}
Lightgbm	{‘lambda_l1’: 0, ‘lambda_l2’: 1, ‘min_data_in_leaf’: 30, ‘num_leaves’: 31, ‘reg_alpha’: 0.1}
Xgboost	{‘colsample_bytree’: 0.3, ‘gamma’: 0.0, ‘learning_rate’: 0.1, ‘max_depth’: 8, ‘min_child_weight’: 1}
STACKB	{use_probas=True, average_probas=False, meta_classifier=Logistic Regresion}
STACKC	{use_probas=True, average_probas=False, meta_classifier=Logistic Regresion}

**Table 5:** Architecture of the custom DNN classifier utilized in this study.

Model: “Sequential”		
Layer (type)	Output shape	Parameters
dense (Dense)	(none, 21)	462
dense_1 (Dense)	(none, 12)	264
dense_2 (Dense)	(none, 9)	117
dense_3 (Dense)	(none, 7)	70
dense_4 (Dense)	(none, 4)	32
dense_5 (Dense)	(none, 1)	5
Total parameters: 950		
Trainable parameters:950		
Non-trainable parameters:950		

**Table 6:** Architecture of the custom 1D-CNN classifier.

Model: “Sequential”		
Layer (type)	Output Shape	Parameters
conv1d (Conv1D)	(none, 21, 32)	128
conv1d_1 (Conv1D)	(none, 21, 64)	6208
conv1d_2 (Conv1D)	(none, 21, 128)	24,704
max_pooling1d (MaxPooling1D)	(none, 11, 128)	0
dropout (Dropout)	(none, 11, 128)	0
flatten (Flatten)	(none, 1408)	0
dense (Dense)	(none, 256)	360,704
dense_1 (Dense)	(none, 512)	131,584
dense_2 (Dense)	(none, 1)	513
Total params: 523,841		
Trainable params: 523,841		
Non-trainable params: 0		

**Table 7:** Summary of results obtained by the DNN and 1D-CNN classifier.

Deep Learning Model	Accuracy (in %)	Precision (in %)	Recall (in %)	F1-Score (in %)	AUC (in %)	Hyperparameters
DNN	87	80	86	83	90	Number of layers: six, neurons: (21,12,9,7,4,1), activation function: relu for first five layers and sigmoid for the last layer, optimizer: adam, loss function: binary cross entropy, batch size: 10, epochs: 1000, learning rate: 0.0001
1D-CNN	90	86	89	88	93	Number of layers: nine, activation function: leaky relu for first eight layers and sigmoid for the last layer, optimizer: adam, loss function: binary cross entropy, batch size: 10, epochs: 200, learning rate: 0.001

## Data Availability

Data cannot be shared since it is obtained from a private hospital after getting ethical clearance.
